# Concise review: current trends on applications of stem cells in diabetic nephropathy

**DOI:** 10.1038/s41419-020-03206-1

**Published:** 2020-11-21

**Authors:** Dongwei Liu, Wen Zheng, Shaokang Pan, Zhangsuo Liu

**Affiliations:** 1 Department of Nephrology, The First Affiliated Hospital of Zhengzhou University, Zhengzhou, 450052 P.R. China; 2 Research Institute of Nephrology, Zhengzhou University, Zhengzhou, 450052 P.R. China; 3 Key Laboratory of Precision Diagnosis and Treatment for Chronic Kidney Disease in Henan Province, Zhengzhou, 450052 P.R. China; 4 Core Unit of National Clinical Medical Research Center of Kidney Disease, Zhengzhou, 450052 P.R. China

**Keywords:** Stem cells, Chronic kidney disease

## Abstract

Diabetic nephropathy, with high prevalence, is the main cause of renal failure in diabetic patients. The strategies for treating DN are limited with not only high cost but an unsatisfied effect. Therefore, the effective treatment of DN needs to be explored urgently. In recent years, due to their self-renewal ability and multi-directional differentiation potential, stem cells have exerted therapeutic effects in many diseases, such as graft-versus-host disease, autoimmune diseases, pancreatic diseases, and even acute kidney injury. With the development of stem cell technology, stem cell-based regenerative medicine has been tried to be applied to the treatment of DN. Related stem cells include embryonic stem cells, induced pluripotent stem cells, mesenchymal cells, and endothelial progenitor cells. Undoubtedly, stem cell transplantation has achieved certain results in the treatment of DN animal models. However, stem cell therapy still remains certain thorny issues during treatment. For instance, poor engraftment and limited differentiation of stem cells caused by the diabetic microenvironment, differentiation into unwanted cell lineages, and malignant transformation or genetic aberrations of stem cells. At present, various researches on the therapeutic effects of stem cells in DN with different opinions are reported and the specific mechanism of stem cells is still unclear. We review here the potential mechanism of stem cells as new therapeutic agents in the treatment of DN. Also, we review recent findings and updated information about not only the utilization of stem cells on DN in both preclinical and clinical trials but limitations and future expectations of stem cell-based therapy for DN.

## Facts

1. Two major mechanisms for the therapeutic effects of stem cell transplantation have been found in DN. One is homing and differentiation and another one is trophic effects.
2. MSCs derived from bone marrow, adipose tissue and umbilical cord blood have been studied extensively in DN both in vivo and in vitro.
3. Tissue-specific iPSCs, such as renal-derived iPSCs, have shown more efficient capacity in differentiated into mature kidney cells.
4. Allogeneic BM-MSCs engraftment has been used in clinical trials on the treatment of DN.

## Open questions

1. What are the specific mechanisms of human umbilical cord extracts WJs improve the therapeutic effect of autologous cell transplantation by improving BM-MSCs abnormalities?
2. Can the stem cells survive under a large environment of diabetes state and maximize their regenerative and repairing effects on DN?
3. Will urine-derived stem cells serve as an effective therapy of DN?

## Introduction

Diabetic mellitus (DM) is considered a chronic, systematic metabolic disease that has been a major cause of death worldwide. According to the World Health Organization, the total number of patients with DM is predicted to 693 million in 2045, only 451 million in 2017^1^. Diabetic nephropathy (DN), as one of the severe microvascular complications of DM, is a leading risk factor for renal failure in patients with end-stage renal disease^2^. Hyperglycemia is a major risk factor for DN, but other characteristics such as glycation end products, overexpression of different growth factors are also related to its pathogenesis^3^. Moreover, high levels of reactive oxygen can induce the generation of inflammatory cytokines in the kidney, which accelerates the progression of DN^4^. However, the mechanism of DN seems more complicated than it appears.

To date, unfortunately, no available treatments can prevent the progression of DN. The current therapeutic strategies are limited to strictly control of hyperglycemia and blood pressure, and the blockade of the RAAS^5^. If required, hemodialysis and kidney transplantation can be the ultimate choices. However, dialysis needs to occupy a lot of time, which seriously affects the quality of patients’ life. In addition, the major dilemma in kidney transplantation for DN is the shortage of renal source and secondary injury of the transplanted kidney in response to high glucose-induced stress. Thus, the development of effective therapeutic strategies is needed imperatively to preserve the renal function and ameliorate the progression of DN.

The kidney is a terminally differentiated organ whose reproductive potential is much lower than other organs in the body. Renal function is not performed by a single cell, but by a unit of cells with different functions, which makes the regeneration of the kidney very difficult. Stem cells have many exploratory studies in various disciplines. Recently, basic research has confirmed that kidney cells have regenerative potential. Stem cells have some exploratory research in treating kidney damage, including DN.

Stem cells are a collection of precursor cells that exist in an undifferentiated state and have an exclusive ability to self-renew. After a long period of asymmetric division, they promote the healthy growth of normal cells due to the polarity of cell division^6^. In addition, stem cells require potency which specifies the potential of differentiating into a variety of specific cell types as another property^7^. Stem cells can be isolated from bone marrow, cord blood, adipose tissue, and several mature adult body tissue including kidney, and even urine^8–10^. Stem cells injected into the body can produce insulin-producing cells, leading to an improvement in blood glucose parameters; or home to the damaged part of the kidney, then differentiate into kidney cells and fuse with damaged cells. Moreover, stem cells can ameliorate renal damage by the pathway of endocrine or paracrine^11^. Growing evidence indicates that replenishing and regulating stem cell therapy represents a multi-faceted contribution to the recovery of diabetic kidney function, thus supporting the use of stem cells as an important therapeutic tool in kidney diseases. In this review, we will discuss the mechanism and potential therapeutic effect of stem cells on DN. Moreover, some major advantages and limitations of all kinds of stem cells and future expectations are also summarized in this article.

## Therapeutic effects of stem cells

Stem cells can be divided into two classes according to the source: embryonic stem (ES) cells and adult stem cells. The ES cells are pluripotent stem cells, which are selected from embryonic inner cell mass or primitive germ cells by inhibiting culture in vitro, have the characteristics of infinite multiplication in vitro, self-renewal, and multi-directional differentiation potential. Theoretically, it can be induced to differentiate into all cell types in the body both in vivo and in vitro. Adult stem cells are classified into extrarenal-derived and intrarenal-derived stem cells, and extrarenal adult stem cells mainly come from bone marrow, umbilical cord blood, adipose tissue, and urine. By far, the most common origination of stem cells in the bone marrow. Bone marrow-derived stem cells can be used to treat acute kidney injury including ischemia/reperfusion^12^ and cisplatin-induced renal damage^13^. It can also alleviate chronic kidney damage, such as streptozotocin (STZ)-induced type I DN^14^ and post-kidney damage caused by 5/6 nephrectomy^15^.

**Fig. 1:**
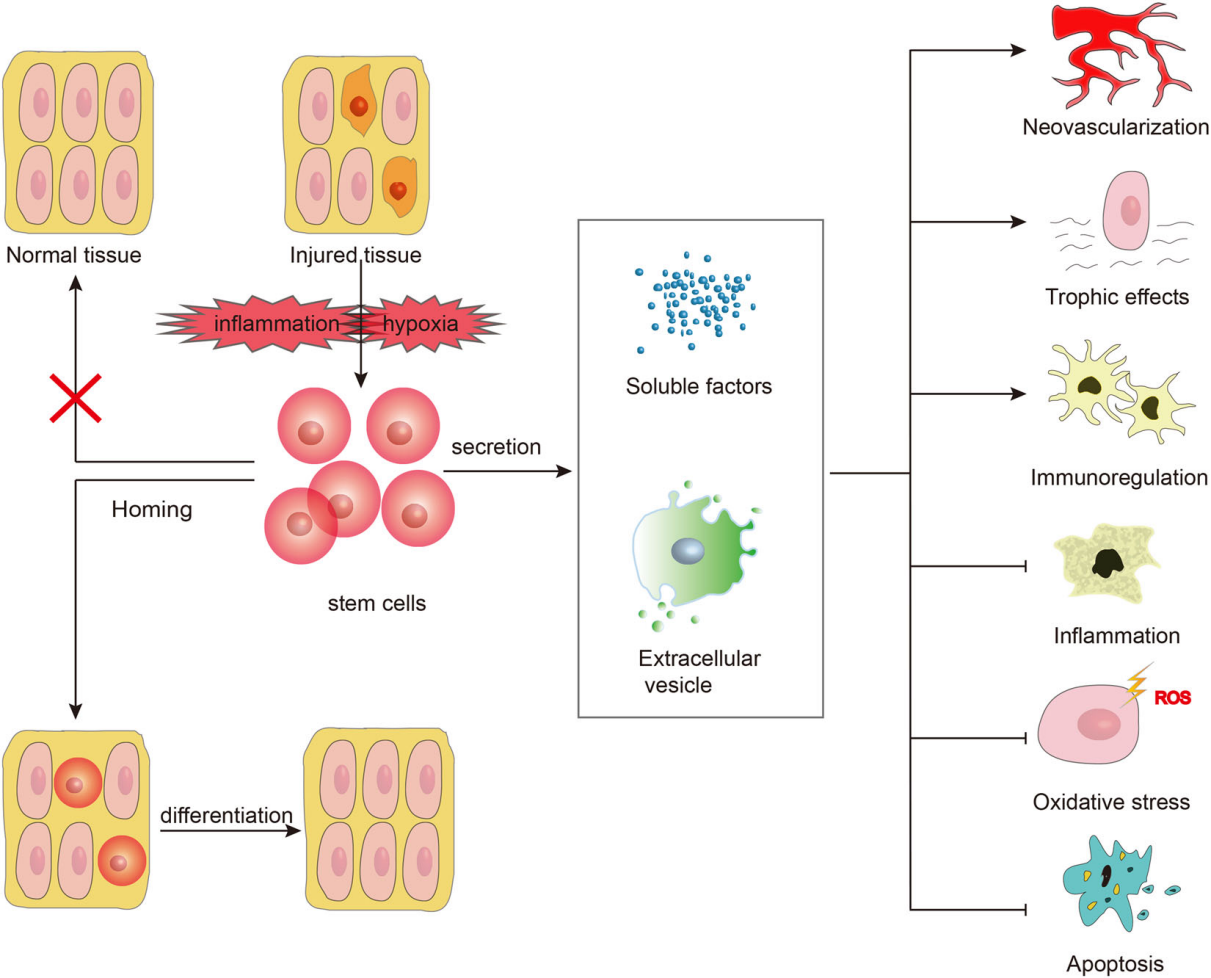
Therapeutic mechanisms of stem cells. Two major mechanisms are involved in the therapeutic effects of stem cell transplantation. One is homing and differentiation. Under the impact of specific environments, such as hypoxia and inflammation in diabetic kidneys, stem cells can recognize the damaged tissues or organs and then home and integrate into specific sites, and finally differentiate into renal tissue cells. The stem cells can only home to the corresponding injured tissue for repair, while this situation does not occur for intact tissues. Another one is trophic effects. Following the secretion of soluble factors and extracellular vesicles, stem cells, especially mesenchymal stem cells, can play a role in protecting renal function and preventing local kidney damage. These secretory factors play therapeutic roles mainly through various mechanisms: neovascularization, trophic effects, immunomodulatory effect, anti-inflammatory, antioxidative stress, and anti-apoptotic effect. Arrow: enhancement; T-bar: reduction.

There are two major mechanisms for the therapeutic effects of stem cell transplantation^16,17^. One is homing and differentiation, which can recognize the damaged tissues and then home and integrate into specific sites, and finally colonize and differentiate into renal tissue cells in specific environmental issues. Another one is trophic effects (Fig. 1). Stem cells play a role by promoting a paracrine, endocrine, or autocrine mechanism such as mitosis, neovascularization, anti-inflammatory, cytoprotective, anti-production, and immune regulation. According to the previous studies, the trophic effect of stem cells on treating DN is inevitable. However, the former effect is a little complicated and thus has recently been debated as an uncertain topic in several fields, especially in the treatment of DN. Wong et al.^18^ have investigated that mesenchymal stem cells (MSC) have the ability to differentiate into glomerular mesangial cells when co-cultured with oxidant-injured mesangial cells in vitro. MSCs can be differentiated into both in vivo and in vitro. Conversely, the Lee group’s results indicate that only a small amount of MSCs was detected in the kidneys of mice after a month of treatment with human MSCs, suggesting that they are unable to proliferate in the kidney^19^. Although there is skepticism about the capacities of stem cells to home to the injured kidney and transdifferentiate into kidney cells, increasing evidence has been supporting this phenomenon, which cannot be ignored. In the following sections, the mechanisms of stem cell therapy will be discussed and summarized.

## Homing and differentiation effect of stem cells

In many studies of stem cell treatment in DN, it has been found that the sufficient homing of stem cells to damaged tissue is extremely important for effective treatment of DN. Previous studies have shown that ES cells can develop into functional renal proximal tubular cells^20^ and mesonephric ducts or ureteric buds^21^. A study, in which four consecutive bone marrow-derived MSCs treatment in the early stage significantly improved renal histology and systemic homeostasis in STZ-induced diabetic nephropathy rats, found that the main location of engrafted MSCs was localized in deterioration areas of the kidneys and immune organs 48 h after infusion^14^. Significant transplantation of stem cells into damaged tissue was observed in these studies but was not in others^22^. For instance, in vitro co-culture experiments showed that human umbilical cord blood-derived MSCs (hUCB-MSC) only inhibited the proliferation of lymphocytes and spleen cells, but did not inhibit mesangial cells. Long-term implantation of hUCB-MSC in the kidney has not been observed either^23^.

When urine-derived stem cells (USCs) were injected into the STZ-induced DM rat model, an enhanced green fluorescent protein (eGFP)-positive USC was observed in the pancreas and kidney, but not in the heart and bladder, indicating that USCs can exert their therapeutic actions by homing into damaged target organs^9^. The state of the tissue may determine whether stem cells can home, fusion, and transdifferentiate or not. In other words, before the stem cells are injected into the body, if the tissue itself is severely damaged, the stem cells will home to the corresponding injured tissue for repair, while this situation does not occur for intact tissues. In stem cells-treated DN mice, normalized β-islet cell regeneration can be observed^24^ and bone marrow-derived MSCs are proved to fused with a small number of parietal epithelial cells^25^. However, there is a very low frequency of the integration of stem cells into uninjured intact tissues. Despite the exact mechanism of stem cells homing to injury, the tissue is still unclear, but hypoxia^26^, inflammation^27^, and high glucose environment^28^ are all present in diabetic kidneys, which can induce migration and proliferation of MSCs.

## Trophic effect of stem cells

Compared with functional recovery after cell transplantation, stem cells have a lower frequency of transplantation and differentiation in different organs, which raises the question of whether homing and differentiation are the main mechanisms of stem cell action. It is widely accepted that stem cells, especially MSCs, can play a role in protecting renal function and preventing local kidney damage via local paracrine effects^29^.

The kidneys of MSC‐treated animals revealed an increase in several growth factors with mitogenic, anti‐apoptotic, and pro‐survival effects, such as insulin‐like growth factor 1 and vascular endothelial growth factor (VEGF) in particular, and a decrease in the expression of pro‐inflammatory cytokines, tumor necrosis factor, interleukin (IL)‐6, and interferon‐gamma (IFN‐γ)^30^. Besides, the intravenous administration of hUCB-MSCs can reduce the production of urinary protein by secreting renal fibronectin and α-smooth muscle actin (α-SMA), and downregulating the expression of E-cadherin^10^. Among the variety of factors produced by stem cells, a number of them are reported to be renoprotective. Several data showed that bone marrow-derived MSCs ameliorated glomerular fibrosis in diabetic rats via secretion of bone morphogenetic protein (BMP)-7^31^ and immunomodulatory cytokines IL-10^32^.

Recent findings suggested that extracellular vesicles, such as exosomes, released by stem cells may also play an important role in the physiological function of these cells^33^. Exosomes are subcellular secretory vesicles with a diameter of about 40–100 nm, which are involved in cell-to-cell communication^34^. Under transmission electron microscopy, the exosomes are flat or globular^35,36^. Exosomes are secreted by a variety of cells and play an imperative role in plasma membrane exchange and transport of biologically active substances such as proteins, messenger ribonucleic acid (mRNA), microRNAs (miRNA), and organelles^37^. A recent study^38^ found that exosomes secreted by human urine-derived stem cells (hUSCs) can prevent kidney damage in type I DN rats. Exosomes from conditioned medium (CM) of urine-derived stem cells (USCs-exo) may contain potential factors, including growth factors, transforming growth factor β1 (TGF-β1), angiopoietin, and BMP7 to reduce high glucose-induced podocyte apoptosis in vitro. Nagaishi et al.^39^ indicated that exosomes purified from MSCs CM have an antiapoptotic effect and maintain the tight junction structure in tubular epithelial cells (TECs), which showed approximately equivalent curative effects with MSCs.

## ES cells

**Fig. 2:**
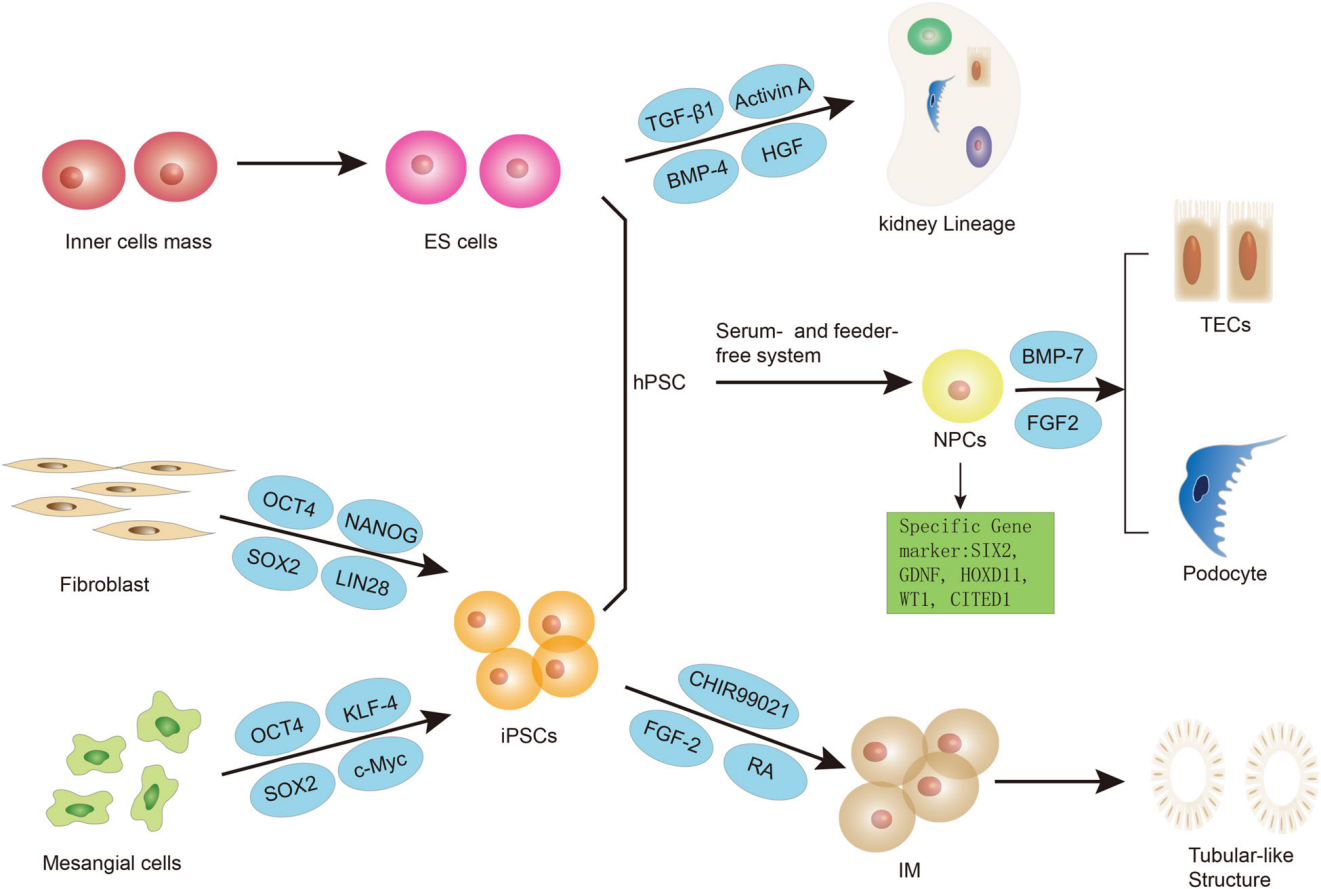
The main differentiated process of ES cells and iPSCs in DN. Embryonic stem (ES) cells are pluripotent stem cells derived from the inner cell mass. Then with the treatment of growth factors such as TGF-β1, activin A, BMP-4, HGF, human ES-derived cells can be differentiated into kidney lineage expressing WT1 and the glomerular marker gene rennin in vitro. Induced pluripotent stem cells (iPSCs) are formed by reprogramming terminally differentiated somatic cells with the assistance of specific transcription factors. Human mesangial cells can be induced into pluripotent stem cells through four defined transcription factors, OCT4, SOX2, KLF4, and c-Myc. Another four factors, OCT4, SOX2, NANOG, and LIN28, are sufficient to reprogram human fibroblasts into iPSCs as well. By chemical induction of small molecule inhibitor CHIR99021 of GSK-3β, human iPSCs can differentiate into intermediate mesoderm (IM) followed by FGF-2 and RA and subsequently form tubular structures upon growth factor withdrawal. In serum and feeder-free conditioned systems, hPSCs (referred to as human ES cells and iPSCs) can differentiate into renal progenitor cells (NPCs) which significantly express the specific gene markers SIX2, GDNF, HOXD11, WT1, and CITED1. Next, NPCs have the potential to differentiate into both tubular epithelial cells (TECs) and glomerular podocytes with BMP-7 and FGF-2 in vitro.

ES cells are pluripotent stem cells derived from inner cell mass in mammalian fertilized eggs^40^. ES cells have been induced in vitro into a wide range of specialized cell types, such as hematopoietic^41^, pancreatic^42^, and neuronal cells^43^. In 1981, the first derivation of mouse ES cells has been developed by Evans and Kaufman^44^ and Martin^45^. It has been reported that injecting wnt4 transformed murine ES cells into adult kidneys can develop into a tubular structure and express AQP-2 on the canal membrane^46^. In vitro, Kim et al.^47^ have found that in combination with renal-derived growth factors such as retinoic acid (RA), activin A, and BMP-7, cultured murine ES cells have the ability to differentiate into intermediate mesoderm (IM) cells. When injected into the developing kidney, the treated ES cells act nearly 100% with renal TECs and can be efficiently integrated into the developing kidney (Fig. 2).

It has been 20 years since the human ES cell line was first reported to have been isolated^48^. Then few reports have depicted the directed differentiation of human ES cells into the kidney lineage. The results indicated that with the treatment of growth factors such as TGF-β1, activin A, BMP-4, and hepatocyte growth factor (HGF), human ES-derived cells can be differentiated into cells expressing Wilms tumor (WT)-1 and the glomerular marker gene rennin in vitro^49^ (Fig. 2). In October 2010, a patient at the Shepherd Center in Atlanta received treatment for the first time with a human ES-based cell therapy product, oligodendrocyte progenitor 1 (OPC1)^50^. Four years later, human ES cells derived islet replacement product, VC-01 was the first type treatment for type I diabetes to enter clinical trials, and the patient was treated at the University of California, San Diego in October 2014^50,51^.

Two limitations of using ES cells are presented. One is the ethical problem of using human fertilized eggs^52^; the other is due to the difference in tissue-compatible antigen between ES cells and patients, and any cell or tissue transplantation has the possibility of immunological rejection^53^. In vitro, murine ES cells have been shown to eliminate allogeneic immune responses and inhibit T cell proliferation by cell contact mechanisms^54,55^. However, the development of proliferative abnormalities, such as teratoma and teratocarcinomas^21^, may still be an important issue for the clinical application of ES cells.

## Induced pluripotent stem cells

Remarkably, differentiation is found to be not a unidirectional process in the past century, and the adult nucleus can restore its developmental potential to a pluripotent state, which is called nuclear reprogramming^53^. Induced pluripotent stem cells (iPSCs), which are pluripotent cells similar to human ES cells, are formed by reprogramming terminally differentiated somatic cells with the assistance of specific transcription factors. In 2006, a breakthrough study^56^ found that four transcription factors commonly found in ES cells, Oct3/4, Sox2, c-Myc, and Klf4, can induce pluripotency in adult mouse fibroblasts, and subcutaneous transplantation of these iPSCs into nude mice resulted in tumors containing various tissues from all three germ layers. Then Yu et al.^57^ reported that another four factors, OCT4, SOX2, NANOG, and LIN28, are sufficient to reprogram human cells into pluripotent stem cells with essential features of ES cells. Undoubtedly, the prospect of obtaining patient-specific pluripotent stem cells from somatic cells is of great interest in the field of regenerative medicine^58^.

Multiple studies have successfully differentiated iPSCs into kidney cells, which may have a potential effect on the treatment of DN (Fig. 2). Bi et al.^59^ firstly reported the iPSCs to differentiate into kidney cells with podocyte characteristics. After 10 days of directed differentiation, iPSCs podocytes upregulated mRNA expression and protein localization of podocyte markers including synaptopodin, renin, and WT-1 while downregulating the stem cell marker OCT3/4. Lam, A. Q. et al.^60^ demonstrated that, by chemical induction of the effective small molecule inhibitor CHIR99021 (CHIR) of GSK-3β, human ES cells and iPSCs (referred to collectively as hPSCs) stably and rapidly differentiate into pluripotent cells expressing intermediate mesodermal markers, which can reproduce the formation of mesoderm in developing embryos followed by fibroblast growth factor-2 (FGF-2) and RA and subsequently form tubular structures upon growth factor withdrawal.

Besides, iPSCs can also differentiate into kidney organoids containing multiple lineages through 3D culture^61^. A study showed that by balancing the anterior–posterior modes of IM with small molecules, human iPSCs can form complex multicellular kidney organoids that contain endothelial cells and renal interstitium. Per organoid consists of more than 500 nephrons and the nephron is similar in transcription to human fetal kidney^62^. In serum and feeder-free conditioned systems, hPSCs can differentiate into renal progenitor cells (NPCs) which significantly express the specific gene markers SIX2, GDNF, HOXD11, WT1, CITED1, and so on. NPCs have the potential to differentiate into both TECs and glomerular podocytes and can form tubular structures in 3D culture systems in vitro^63^. A simple and effective method for obtaining functional podocytes from human iPSCs in vitro was reported in another study^64^. The cells were exposed to a three-step therapy, induced to differentiate into mesodermal layers, then differentiated into renal progenitor cells, and finally into mature podocytes. These podocytes are capable of internalizing albumin and self-assembly into a chimeric three-dimensional structure by binding to isolated mouse embryonic kidney cells^64^.

Tissue-specific iPSCs, such as renal-derived iPSCs, maybe more efficiently differentiated into mature kidney cells than unrelated tissue iPSCs or even ES cells^65^. Song et al.^66^ successfully induced human mesangial cells into pluripotent stem cells through four defined transcription factors, OCT4, SOX2, KLF4, and c-Myc, and confirmed that iPSCs have similar biological morphology and gene expression to ES cells. And it is possible for iPSCs to form embryoid bodies and express markers of three germ layers. In another study, they obtained iPSCs from chronic kidney disease patients undergoing hemodialysis due to diabetic nephropathy and glomerulonephritis (HD-iPSCs), and HD-iPSCs derived NPCs exhibited the possibility of maturation and vascularization in vivo when transplanted into mice, similar to NPCs derived from healthy controls. These findings suggested that HD-iPSCs have sufficient ability to differentiate into functional nephrons in vivo^67^. In addition, researchers have successfully generated iPSCs by using kidney tubular cells in urine and confirmed their multiple differentiation potential through directional differentiation of neuronal cells, cardiomyocytes, and hepatocytes corresponding to three germ layers respectively^68^.

A sufficient number of starting cells is critical to treat DN. In order to reduce the difficulty of that directed differentiation of human iPSCs from a specific type of mature kidney cells with low yield, Musah et al.^69^ established a protocol that can use a microfluidic organ-on-a-chip culture device to induce directed differentiation with high efficiency (>90%). Besides, the cells in the urine are easy to collect and expand, Zhou et al.^68^ have found a way to solve the problems encountered in the production of iPSCs. The directed differentiation of iPSCs to a particular lineage appears to reproduce the naturally occurring developmental state of the embryo. However, compared to cells obtained from ES cells, studies have represented that differentiated cells isolated from human iPSCs show a possibility of premature aging, decreased efficiency, and increased variability, which raised concerns about somatic-derived recombinant cells^70,71^.

## Mesenchymal stem cells

MSCs were first discovered and extracted from the bone marrow by A.J. Friedenstein as early as the 1960s^72^. MSCs are more pluripotent than normal somatic cells which presented as they can not only differentiate into adipocytes, osteoblasts, chondrocytes, but into non-mesodermal cells such as blood vessels, kidneys, and skin tissues both in vitro and in vivo^24,73^. However, unlike typical pluripotent stem cells, such as EC cells, MSCs do not indefinitely self-renew and are not homogeneously pluripotential^74^. In fact, MSCs can be found in a variety of tissues and organs, including bone marrow, adipose tissue, lung, kidney, liver, brain, and umbilical cord blood^75^. The current research showed that MSCs can be implanted into the kidney, differentiate into kidney cells, improve renal function, and regeneration of glomerular structure, thus effectively treating DN^19,24^. In this section, we will focus on the application of MSCs isolated from three major issues in the treatment of DN (Table 1).

**Table 1:** Preclinical studies of mesenchymal stem cell therapy on animal models of diabetic nephropathy.

MSC types	Experimental models	Treatment		Effects on DN	Reverse the hyperglycemia or not	Reference
		Injection methods	Frequency and Dose			
*BM-MSCs*						
Human BM-MSCs	STZ-induced type I NOD/SCID mice	Intracardiac infusion	Days 10 and 17 2.5 × 10^6^	➢ Pancreatic islets and cells producing mouse insulin has increased ➢ A decrease in mesangial thickening and in macrophage infiltration	Yes	Lee et al.^19^
Mice BM-MSCs	STZ-induced type I C57BL/6 mice	Tail vein injection	Two doses (interval of 21 days) 0.5 × 10^6^	➢ MSC-treated mice showed only slight tubular dilatation ➢ Small number of donor cells homed and persisted in the kidney	No	Ezquer et al.^78^
	STZ-induced type I C57BL/6 mice	Tail vein injection	Single dose 0.5 × 10^6^	➢ Increase in morphologically normal beta-pancreatic islets ➢ Ameliorate glomerular hyalinosis and mesangial expansion	Yes	Ezquer et al.^24^
Rat BM-MSCs	STZ-induced type I female Wistar rats	Tail vein injection	Once a week for twice 2 × 10^6^	➢ Inhibit TGF-β/Smad signaling pathway ➢ Secreted a significant amount of BMP7 ➢ Attenuate the renal function and the glomerulosclerosis	Yes	Lv et al.^31^
	STZ-induced type I Sprague Dawley rats	Tail vein injection	Single-dose (4 weeks after the onset) 2 × 10^6^	➢ Inhibit TGF-b1/Smad3 pathway ➢ Decrease the expression of PAI-1 ➢ Reduce the accumulation of extracellular matrix ➢ Inhibit renal fibrosis in rats with DN	No	Lang et al.^79^
	STZ-induced type I Sprague Dawley rats	Tail vein injection	Single-dose (4 weeks after the onset) 3 × 10^6^	➢ BMSCs differentiate into islet-like cells with miR-124a ➢ MSCs combined with miR-124a enhance proliferation and inhibit apoptosis of podocytes ➢ Protected kidney tissue from impairment and inhibit nephroncyte apoptosis	Yes	Sun et al.^81^
	STZ-induced type I Sprague Dawley rats	Tail vein injection	Four doses (2, 4, 5, and 7 weeks after the onset) 5 × 10^6^	➢ Upregulate serum anti-inflammatory cytokines IL-10 and EGF ➢ Downregulate inflammatory-related cytokines such as IL-6, MCP-1, TNF-α, and IL-1β ➢ Engrafted MSCs were primarily localized in deteriorated areas of the kidney and immune organs	No	Li et al.^14^
	STZ-induced type I female Wistar rats	Tail vein injection	Once a week for twice 2 × 10^6^	➢ Suppress the expression of MCP-1 and the number of infiltrated macrophages in the kidney ➢ Up-regulate the expression of HGF ➢ Downregulate the expressions of IL-1β, IL-6, and TNFα	Yes	Lv et al.^83^
	HFD-induced type 2 C57BL/6J mice STZ-induced type I C57BL/6 J mice	Tail vein injection	1.0 × 10^4^ MSCs/g (4 times every 2 weeks) for HFD-induced mice 1.0 × 10^4^ MSCs/g (2 times every 4 weeks) for STZ-induced mice	➢ Inhibit the exacerbation of albuminuria ➢ Inhibit the increase of glomerular mesangium substrate in HFD diabetic mice ➢ Exosomes purified from MSC-CM exert an anti-apoptotic effect and protect tight junction structure in TECs	No	Nagaishi et al.^39^
	STZ-induced type I Sprague Dawley rats	Tail vein injection	Single-dose with ultrasound-targeted microbubble destruction (UTMD) technique 1 × 10^6^	➢ UTMD increase the permeability of renal interstitial capillaries and VCAM-1 expression ➢ Inhibit TGF-β1 expression and upregulate synaptopodin and IL-10 expression	Yes	Yi et al.^32^
	STZ-induced type I Sprague Dawley rats	Left renal artery injection	Single dose 2 × 10^6^	➢ Suppress creatinine clearance rate and urinary albumin to creatinine ratio ➢ Reduce the dysfunction of podocytes ➢ Express higher levels of BMP-7 but not of VEGF	No	Shuai et al.^86^
Tree shrew BM-MSCs	STZ-induced type I tree shrews	Intravenous injection	Two doses (interval of 14 days) 5 × 10^6^	➢ Biological indexes were significantly lowered ➢ Home to the kidney and pancreas of DN tree shrews	Yes	Pan et al.^80^
*AD-MSCs*						
Human AD-MSCs	STZ-induced type I Sprague Dawley rats	Tail vein injection	Five doses (at 4 weekly intervals) 5 × 10^6^	➢ Attenuate glomerulus hypertrophy and tubular interstitial jury ➢ Downregulate the expression of WT -1 and synaptopodin ➢ The cells were detected in lung, spleen, and peritubular regions, but rarely in the pancreas	No	Zhang et al.^96^
Autologous AD-MSCs	STZ-induced type I Sprague Dawley rats	Tail vein injection	Single-dose (4 weeks after the onset) 1 × 10^7^	➢ Minimize pathological alterations, reduce oxidative damage, and suppress the expression of pro-inflammatory cytokines ➢ Decrease molecules of the MAPK signaling pathway.	Yes	Fang et al.^97^
Rat AD-MSCs	STZ-induced type I Sprague Dawley rats	Tail vein injection	Single dose 1 × 10^7^	➢ Reduce the rate of cellular apoptosis ➢ Decrease Bax and Wnt/β-catenin levels ➢ Elevate Bcl-2 and klotho levels	Not Mentioned	Ni et al.^92^
*UCB-MSCs*						
Human UCB-MSCs	STZ-induced type I Sprague Dawley rats	Tail vein injection	Single-dose (4 weeks after the onset) 1 × 10^6^	➢ Reduce proteinuria and renal fibronectin ➢ Up-regulate α-SMA and down-regulate renal E-cadherin ➢ Engraft human UCB-MSC in diabetic kidneys	No	Park et al.^10^
	STZ-induced type I Sprague Dawley rats	Tail vein injection	Single dose 5 × 10^5^	➢ A few engraftments of hUCB-MSC in diabetic kidneys ➢ hUCB-MSC conditioned media inhibit TGF-b1-induced extra-cellular matrix upregulation and epithelial-to-mesenchymal transition ➢ Prevent diabetic renal injury	No	Park et al.^16^

## Bone marrow-derived MSCs

Bone marrow-derived MSCs (BM-MSCs) have a wide range of functions, besides being able to differentiate into specific cells, they can also improve the tissue microenvironment by secreting a variety of factors, such as growth factors, interleukins, chemokines, and repair local damaged tissue through paracrine role^76,77^. In preclinical models, the therapeutic effects and mechanisms of BM-MSCs on DN have been extensively studied. Previous studies showed that intravenous injection of BM-MSCs not only prevents renal failure ameliorates glomerular fibrosis in C57/BL diabetic mice^31,78^ but also promotes repair of renal glomerular and islet cells injury in diabetic NOD/SCID mice^19^. Besides, BM-MSCs significantly inhibited renal fibrosis in Sprague Dawley rats with DN by reducing the accumulation of extracellular matrix, thereby balancing the fibrinolytic system^79^. In one study, a tree shrew DN model was successfully established for the first time by a high-sugar and high-fat diet and four injections of STZ. BM-MSCs were delivered to the kidneys and pancreas of DN tree shrews through the tail vein, and BM-MSCs were found to significantly improve the kidney damage of DN in tree shrew^80^. However, there are few studies related to the therapeutic effects of systemic administration of BM-MSCs on type 2 DN animals.

A study demonstrated that miR-124a can promote the differentiation of BM-MSCs into islet-like cells, thus prevent the damage of renal tissue and reduce renal cell apoptosis in DN by inhibiting the Notch signaling pathway^81^. However, the administration of BM-MSCs for targeting live animal tissues has proven to be limited. For instance, MSCs engraftment typically results in an insufficient number of MSCs transplanted at the site of injury^82^. Therefore, the role of BM-MSCs is primarily a result of the paracrine mechanism initiated by transplanted cells. Li et al.^14^ intervened BM-MSCs four times every two weeks in the early stage of the onset of STZ-induced diabetic rats. It was found that BM-MSCs can significantly improve renal histology and systemic homeostasis by upregulating the serum levels of anti-inflammatory cytokines, such as epidermal growth factor (EGF) and IL-10, thus prevent renal insufficiency and glomerular sclerosis. Moreover, BM-MSCs can improve the glomerular injury in STZ-induced DN rats by secreting HGF and reducing the expression of MCP-1 to inhibit macrophage infiltration^83^ or by inhibiting oxidative stress^84^. Particularly, some studies found BM-MSCs achieve paracrine functions by secreting exosomes^39^.

Current evidence suggests that improved glycemic control can significantly delay the development and progression of DM complications^85^. Transplantation of BM-MSCs can reverse hyperglycemia and may be associated with the regeneration of beta islets^24,32^. However, BM-MSCs have no significant effect on the level of hyperglycemia in many studies^78,79,86^. In order to avoid immune rejection caused by allogeneic bone marrow transplantation, one of the key solutions of MSC therapy is the isolation of MSCs from diabetic patients for autologous transplantation. Nagaishi et al.^87^ developed a new method for improving BM-MSCs abnormalities with human umbilical cord extracts named WJs, which can significantly improve the therapeutic effect of autologous cell transplantation, but the specific mechanism remains unclear. Moreover, obtaining bone marrow is an invasive process, and the decrease of the number and functional incompetence of BM-MSCs may be caused by the toxicity of uremia^88^ and age^89^.

## Adipose tissue-derived MSCs

Adipose tissue-derived MSCs (AD-MSCs) are multipotent stem cells with similar characteristics to BM-MSCs, derived from the vascular-stromal compartment of adipose tissue^90,91^. The expression of cluster of differentiation (CD) 90^+^ on the surface of ADMSCs is abundant, but the expression of CD13^+^ and CD45^+^ is less^92^. ADMSCs are capable of differentiating into several lineages of mesoderm origin, including fat, cartilage, and osteoblasts. And AD-MSCs can be used as an alternative therapy to many diseases because of their self-renewal and differentiation into different cell types^93^. Ni et al.^92^ found that transplantation of AD-MSCs can ameliorate renal damage in STZ-induced DN rats through activating Klotho and inhibiting Wnt/β-catenin pathway. In addition, AD-MSCs can also prevent high glucose-induced podocyte apoptosis and injury mainly by secreting soluble EGF. Studies have found that AD-MSCs CM improves the reduction and disorder of synaptic peptides and renin in podocytes, which may relate to the secretion of EGF via exosomes^94,95^. Repeated systemic injection of human AD-MSCs has been proved to alleviate renal injury in DN rats by paracrine trophic factors rather than differentiation^96^. However, the optimal dose and frequency of AD-MSCs transplantation for DN are still under debate. There is reported for the first time, autologous transplantation of AD-MSCs significantly reduced blood glucose levels in STZ-induced rats and protected rats from DN by inhibiting oxidative stress, the release of pro-inflammatory factors, and p38-MAPK pathway^97^. Unlike BM-MSCs, human adipose tissue is relatively easy to obtain, and there are fewer lesions at the donor site and fewer patients’ discomfort. Therefore, the administration of autologous AD-MSCs is safe and is expected to be used for the treatment of DN.

## Umbilical cord blood-derived MSCs

Umbilical cord blood-derived MSCs (UCB-MSCs), extracted from cord blood that was once considered as medical waste, are currently used as a substitute for bone marrow as a source of stem cells for the treatment of various diseases^10,98^. UCB-MSCs have the advantages of easy procurement, immediate availability, and possess the better proliferative potential and lower graft-versus-host disease and teratogenic risk than BM-MSCs^99^. Masoad et al.^100^ found that monocyte stem cells derived from umbilical cord blood have a therapeutic effect on STZ-induced diabetic rats. After 4 weeks of DN, the administration of human UCB-MSCs in the tail vein, under the condition of persistent hyperglycemia, can alleviate renal impairment of DN^10^. Thus, they proposed that UCB-MSCs have a role of direct renoprotection. However, transplantation of human umbilical cord blood mononuclear cells can alleviate high blood glucose and glomerular hypertrophy in type 2 DN mice^101^. Park et al.^16^ demonstrated hUCB-MSCs CM attenuated the mRNA expression of TGF-β1, α-SMA, collagen I, and heat shock protein(HSP)-47, meanwhile increased the mRNA expression of E-cadherin and BMP-7, indicating hUCB-MSCs can prevent kidney damage in DN rats through paracrine humoral factors. Therefore, the treatment and improvement of renal function in mice with hUCB-MSCs are mainly due to immunomodulatory effects, rather than local implantation or differentiation into kidney cells^23^.

## Urine derived-stem cells

Researchers isolated and amplified a subpopulation of cells from 55 urine samples, which possess characteristics of progenitor cells and have the potential to differentiate into several bladder cell lineages, known as urinary derived stem cells (USCs)^102^. USCs are more homologous to the urinary system and are capable of differentiating into endothelial cells, osteoblasts, chondrocytes, adipogenesis, and neurogenic lineage but did not form teratomas within 1-month study^103^. In the present study, a double-dose injection of USCs via the tail vein, rather than urethra can effectively improve the impaired renal injury not only via homing to damaged organs such as the pancreas and kidneys but secreting many pro-angiogenic factors^9^. After gene modification with FGF-2, USCs was founded to improve the complications of type 2 DN rats by secreting pro-angiogenic trophic factors and immunoregulators in vitro^104^. In addition, exosomes secreted by USCs may prevent diabetic kidney damage by inhibiting podocyte apoptosis and promoting angiogenesis and cell survival^38^. A major advantage of USCs is that these cells can be obtained noninvasively in large quantities and amplified extensively in vitro^105^ so that a sufficient number of cells is readily available to treat the disease. However, the definite mechanism of USC’s treatment of DN remains unclear, and further researches are needed.

## Endothelial progenitor cells

Endothelial progenitor cells (EPCs) are derived from CD34^+^ hematopoietic stem cells, which can differentiate into red blood cells, platelets, various leukocyte lineages, and endothelial cells by different pathways^106,107^. Different markers were used to describe circulating EPCs in vivo, including CD34^+^ kinase-insert domain receptor (KDR^+^), CDl33^+^KDR^+^, and CD34^+^CD133^+^KDR^+^^108^. EPCs are helpful to maintain vascular homeostasis and play an important role in maintaining vascular endothelial function. For example, some researchers have observed that EPCs are mobilized to damaged glomeruli and may be directly involved in the regeneration of glomerular capillaries^109^. Recent studies have shown that the reduction in EPCs may also be a causative factor in microvascular disease, as a significant association with clinical manifestations has been found in retinopathy^110^, nephropathy^111^, and wound healing^112^. Current studies about the impact of diabetes status on EPCs and the therapeutic effects of EPCs in DN will be described in the continued sections.

## The effect of diabetes on EPCs

**Fig. 3:**
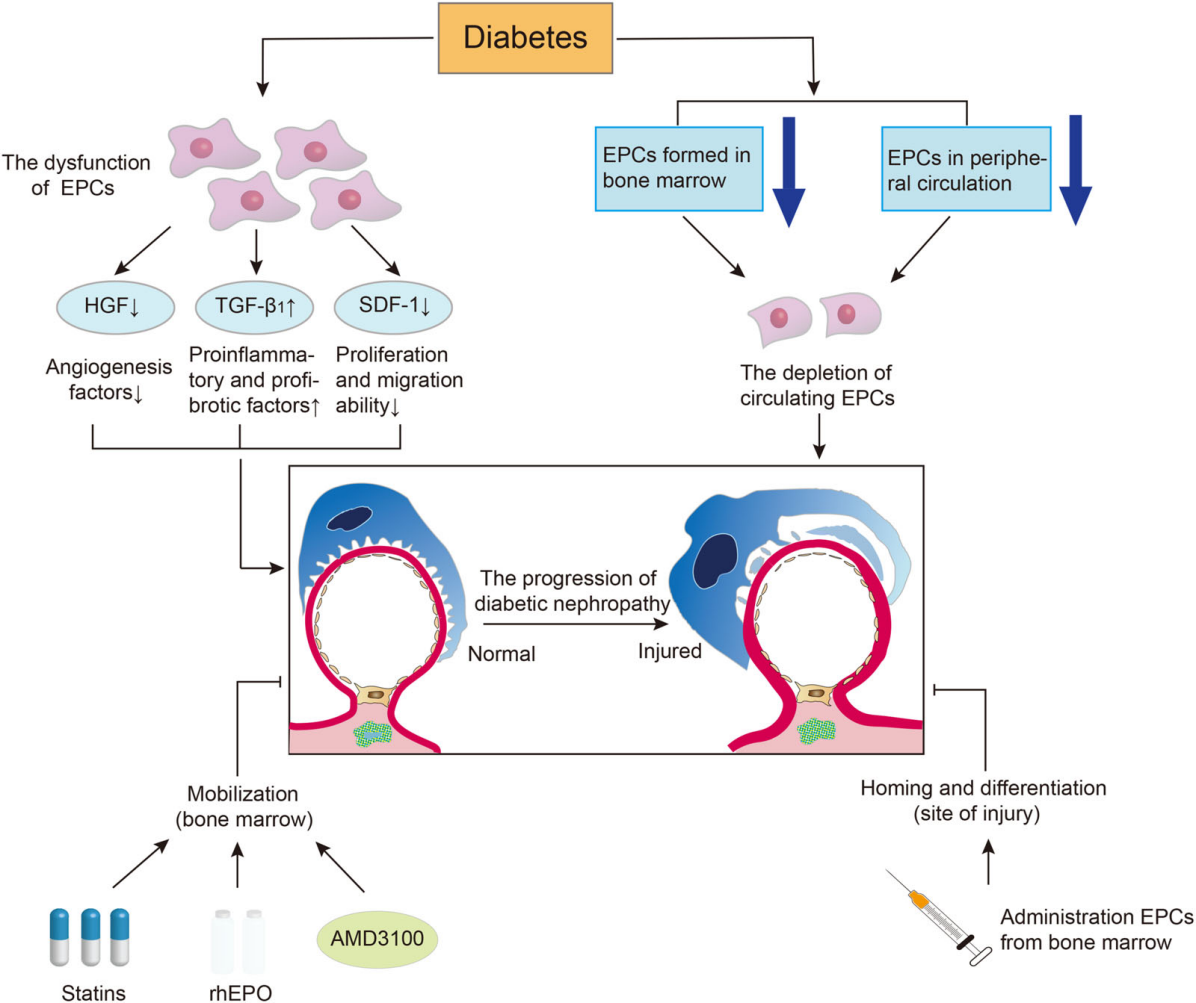
The effect of diabetes on EPCs and the application of EPCs in DN. In diabetic conditions, the paracrine function of endothelial progenitor cells (EPCs) are damaged, whereupon the expression of angiogenesis factors in EPCs and the proliferation and migration ability of EPCs is reduced, while the secretion of proinflammatory and profibrotic factors such as TGF-β1 is increased. Moreover, the depletion of circulating EPCs is due to a decrease of EPCs formed in bone marrow and EPCs in the peripheral circulation. Decreased circulating levels of EPCs and functional disorder of EPCs in diabetes may lead to the progression of diabetic nephropathy (DN). Conversely, the administration of statins, recombinant human erythropoietin (rhEPO), and AMD3100, an effective EPCs mobilizer, can accelerate the healing of wounds in diabetic patients by promoting EPCs mobilization in the bone marrow. In addition, the injection of bone marrow-derived EPCs are able to repair the glomerular endothelial injury and might achieve the purpose of treating DN. Arrow: acceleration; T-bar: amelioration.

To date, decreased levels of circulating EPCs and functional disorder of EPCs have been reported in diabetes, suggesting that circulating EPCs may lead to DM vascular complications^113^ (Fig. 3). On the one hand, diabetes is characterized by microvascular and macrovascular lesions, manifesting dysfunction of EPCs. The paracrine function of EPCs has been found damaged in diabetic conditions. Compared with non-diabetes, the proliferating angiogenesis factors such as HGF secreted by CD34^+^ cells of diabetes are reduced, while the secretion of proinflammatory and profibrotic factors such as TGF-β1 is increased, and meanwhile, the proliferation and migration ability of EPCs which can be responded by stromal-derived factor-1 is reduced^114^. Leicht et al.^115^ found that EPCs isolated from type 2 diabetic patients showed impaired cell proliferation and migration compared to counterparts isolated from young healthy donors or age-matched nondiabetic subjects. On the other hand, the depletion of EPCs may also be a causative factor in diabetic microangiopathy. In diabetic conditions, the depletion of circulating EPCs is due to a decrease of EPCs formed in bone marrow and EPCs in the peripheral circulation^116,117^. A study demonstrated that urinary albumin excretion rate increased after 1 year in patients who were diagnosed with reduced EPCs, indicating a relationship between EPCs reduction and DN progression^111^.

## Application of EPCs in DN

Based on the above characteristics of EPCs, in theory, as long as it is a method that can protect the number and function of EPCs, it may become a treatment for diabetic vascular disease (Fig. 3). A study found that the mobilization of EPCs to vascular injury in type 1 diabetic rats was limited, and the mobilization of EPCs was to some extent restored after treatment with insulin and related cytokines, suggesting that mobilization of EPCs is sensitive to hyperglycemia, and early treatment is reversible^118^. Statins, such as atorvastatin, can promote EPC mobilization in the bone marrow and increase circulating EPC in the blood under diabetic states without relying on the role of lowering cholesterol^116^. A series of studies showed that administration of recombinant human erythropoietin (rhEPO) or its analog darbepoetin alfa can participate in the process of repairing the cardiovascular system both in vivo and in vitro^119,120^. After 4 weeks of injection, darbepoetin alfa increased the number of EPCs subgroups, improved endothelial function and vascular reactivity, and increased endothelial cell proliferation^120^. Moreover, the administration of AMD3100, which has been considered as an effective EPCs mobilizer, can promote the healing of wounds by mobilizing EPCs in diabetic patients^121^. In addition, a previous report indicates that injection of bone marrow-derived EPCs can improve glomerular endothelial injury and mesangial activation in the AGN model, suggesting that EPCs are able to repair glomerular endothelial injury^122^. By regenerating damaged endothelial cells and repairing blood vessels, EPCs might achieve the purpose of treating DN.

## Conclusion and future expectations

With the advancement of stem cell technology and application level, the use of stem cells to reconstruct and repair damaged kidney tissue will greatly promote the treatment of DN. In recent years, stem cell-based therapies have been studied more in detail and promising results were acquired by administrating various types of stem cells in preclinical models. In this article, we summarized stem cells that can currently be used to treat DN, including ES cells, iPSCs, and somatic stem cells, which can play a therapeutic role on DN by homing to the injured sites of tissues and organs and then differentiating into specific tissues or by secreting various trophic factors. In turn, diabetes also affects the ability of stem cells, such as migration, proliferation, and differentiation. No matter what sources are stem cells derived from, it will have certain challenges, such as how to solve the low induction rate of somatic cells, and how to make a large number of differentiated stem cells more effectively distributed in the damaged kidney, remain to be further studied.

One of the difficulties in stem cell therapy is the low survival rate of transplanted cells stimulated by high glucose. Endogenous factors can cause cell death through excessive oxidative stress and mitochondrial damage. And the uremic state will reduce the expression of VEGF in stem cells and accelerate cell senescence, resulting in insufficient angiogenesis. Therefore, managing to survive stem cells under a large environment of diabetes states and maximizing their regenerative and repairing effects may be a bright prospect for treating DN. In the current stem cell research, the safety and efficacy of BM-MSCs are the most in-depth study. However, most studies on MSCs are focused on type I diabetes, and there is still rare research on type 2 DN caused by obesity or insulin resistance. Besides, stem cell therapy for DN is still limited to studies of preclinical models. So far, there is only one clinical application of stem cells in DN patients. In October 2015, a phase I/II clinical trial (NCT02585622) was conducted on the treatment of DN with allogeneic BM-MSCs engraftment, and the preliminary efficacy of BM-MSCs was confirmed in terms of safety, feasibility, and tolerability^123^. Based on successful preclinical experiments in the treatment of DN animals with stem cells, what we should do is to conduct further clinical trials to validate the efficacy of stem cell therapy in the treatment of DN. In brief, we hope that future experiments are not only for merely mechanistic insights but also for the development of new curative strategies.
